# Clinical, Imaging, and Genetic Characteristics of Adult Left Ventricular Noncompaction: A Single-Center Registry Study

**DOI:** 10.31083/RCM54341

**Published:** 2026-10-14

**Authors:** Ava Travo, Pratham Gupta, Megan Smith, Mobeen Ahmed, Ahmed T. Moustafa, Habib Rehman Khan

**Affiliations:** ^1^London Health Sciences Center, University of Western Ontario, London, ON N6A 3K7, Canada; ^2^Department of Medical Imaging, London Health Sciences Centre, Schulich School of Medicine, Western University, London, ON N6A 5A5, Canada; ^3^Division of Cardiology, Western University, London, ON N6A 5A5, Canada

**Keywords:** trabeculation, myocardial compaction, noncompacted-to-compacted ratio, late gadolinium enhancement, premature ventricular complexes, risk stratification

## Abstract

**Background:**

Left ventricular noncompaction (LVNC) is a heterogeneous cardiomyopathy characterized by excessive myocardial trabeculation and a variable clinical presentation. Although advances in multimodality imaging have improved diagnostic accuracy, prospective longitudinal risk stratification frameworks remain limited.

**Methods:**

This single-center registry study describes the baseline clinical, imaging, electrocardiographic, and genetic characteristics of adult patients with confirmed LVNC followed at a tertiary inherited cardiac conditions clinic, enrolled both prospectively and retrospectively. Baseline assessments included demographics, clinical history, transthoracic echocardiography (TTE), cardiac magnetic resonance imaging (CMR), electrocardiography, exercise stress testing, Holter monitoring, and genetic testing. This descriptive analysis does not include a formal time-to-event outcomes analysis, which is planned for future prospective follow-up of this cohort.

**Results:**

The cohort comprised 50 adults (34 women, 16 men; mean age at diagnosis 48.9 ± 13.4 years), enrolled prospectively (n = 38) and retrospectively (n = 12). Diagnosis was established by CMR in 80% of patients (n = 40), TTE in 18% (n = 9), and CT in 2% (n = 1). Presenting features included prior cardiac arrest (n = 3), syncope (n = 10), palpitations (n = 16), and a family history of sudden cardiac death (n = 9). Baseline TTE (available in 38 patients) showed a mean left ventricular ejection fraction (LVEF) of 51.8 ± 11.8%. CMR, available for 44 patients (88% of the cohort), confirmed LVNC in all cases, with the noncompacted-to-compacted ratio (NC:C) ranging from 2.8:1 to 6.3:1 (median 4.0:1); apical hypertrabeculation was universal, and lateral involvement was present in 72.7% (32/44) of CMR-imaged patients. Among 48 patients with electrocardiogram (ECG) data, T-wave abnormalities were present in 20.8% (10/48), left bundle branch block in 14.6% (7/48), and right bundle branch block in 6.3% (3/48). Genetic testing, performed in 32 of the 38 prospectively enrolled patients, identified pathogenic or likely pathogenic variants in 53% (17/32), most commonly in TTN (34%, 11/32); the remainder had variants of uncertain significance or no identified variant. Structured follow-up data were incomplete and variably attended, with follow-up 2 assessments available in 29 of 50 patients (58%); among those retested, LVEF remained preserved or only mildly reduced (mean 51.1% and 56% at follow-up 1 and 2, respectively).

**Conclusions:**

This single-center registry demonstrates substantial phenotypic, morphologic, and genetic heterogeneity in adult LVNC, with CMR providing the most sensitive morphologic characterization and pathogenic or likely pathogenic variants identified in approximately half of genetically tested patients. Since clinical outcome events and time-to-event analyses were not assessed in this baseline dataset, these findings are hypothesis-generating rather than prognostic. Thus, structured prospective follow-up of this cohort is needed to define arrhythmic and heart failure outcomes and to refine phenotype- and genotype-directed risk stratification.

**Clinical Trial Registration:**

NCT06024759 (https://clinicaltrials.gov/study/NCT06024759?cond=NCT06024759&viewType=Card&rank=1).

## 1. Introduction

Left ventricular (LV) noncompaction (LVNC), also known recently as LV hypertrabeculation, is a heterogeneous myocardial disorder characterized by excessive trabeculations of the LV [1,2]. LVNC results from arrested embryonic myocardial compaction, which normally occurs between weeks 5–8 of gestation, leaving a two-layered myocardium with prominent trabeculations and deep intertrabecular recesses [3]. The compaction process transforms a loose meshwork of interwoven fibres known as a trabecular network into the characteristic two-layered myocardium for the formation of a mature heart, with a compacted epicardial layer and a thick noncompacted endocardial layer [1,4].

An important distinction, increasingly emphasized in the literature, is between LVNC as a distinct cardiomyopathy and LV hypertrabeculation as a morphologic trait that can be found incidentally, in athletes, in pregnancy, and in other loading conditions without an underlying myocardial disease. Excess trabeculation alone, in the absence of ventricular dysfunction, arrhythmia, or a corroborating family or genetic history, does not necessarily indicate a pathological cardiomyopathy, and imaging criteria for LVNC have been met by a substantial proportion of healthy individuals. This distinction has direct clinical relevance, since labelling isolated hypertrabeculation as a cardiomyopathy risks overdiagnosis, unnecessary restriction, and inappropriate device therapy, whereas underrecognizing true LVNC cardiomyopathy in a patient with ventricular dysfunction or a high-risk genotype risks missing arrhythmic and heart failure risks [5,6].

Despite growing recognition of LVNC, most published cohorts are limited by variable diagnostic criteria, small genetically characterized subgroups, and short or incomplete follow-up, so the relationship between morphologic severity, genotype, and clinical course remains poorly defined. To address this gap, we established a single-center registry of adult LVNC patients at the Inherited Cardiac Conditions (ICC) Clinic, University Hospital, London Health Sciences Centre, integrating standardized clinical, multimodality imaging, electrophysiologic, and genetic data. This report presents the baseline characterization of that registry, describing the demographic, imaging, electrocardiographic, and genetic features of the cohort as a foundation for planned prospective outcome analyses.

### 1.1 Patient Demographics and Risk Profile

This diagnostic evolution is further highlighted by emerging technology, such as population-based cardiac magnetic resonance (CMR). Recent CMR studies have revealed substantially higher estimates than previously reported; for instance, 14.8% of healthy individuals met at least one diagnostic criterion for LVNC, while 4.4% met the most specific criteria [7]. This stands in stark contrast to other studies that reported a significantly lower prevalence, ranging from 0.014% to 0.26% in the general population [2,8]. Such a dramatic increase in identified cases underscores the urgent need for a more holistic understanding of LVNC risk factors and their subsequent morbidities. Distinguishing between benign morphological traits and true pathological states is essential to improve the diagnosis, treatment, and long-term management of patients.

### 1.2 Diagnostic Imaging Criteria of LVNC

#### 1.2.1 Echocardiography Imaging Criteria

LVNC is primarily diagnosed using non-invasive imaging, namely two-dimensional transthoracic echocardiography (TTE), due to its accessibility, cost-effectiveness, and thus serves as the primary diagnostic tool for LVNC [1]. This modality allows clinicians to identify the hallmark structural features of the condition: a distinct, two-layered myocardium characterized by extensive trabeculations and deep intertrabecular recesses that open directly into the LV cavity (Fig. 1).

**Fig. 1.**
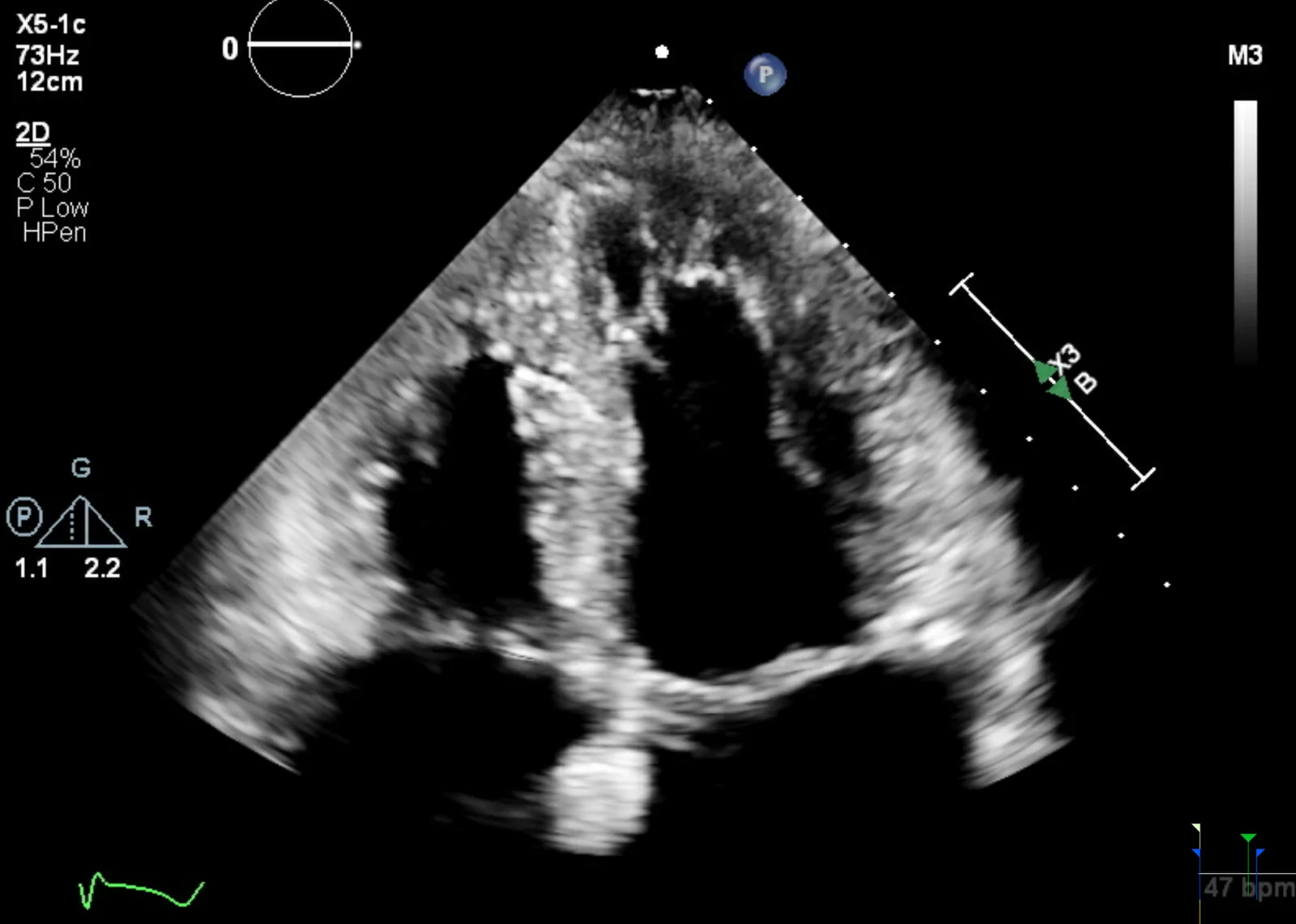
**TTE of the left ventricle, showing apical-lateral hypertrabeculations**. Parasternal short axis, 2D, diastole. TTE, transthoracic echocardiography.

Diagnosing LVNC on an echocardiogram requires assessment of left ventricular free-wall thickness at end-diastole, prominence of trabeculations, and calculation of the LVNC trabeculation assessment (X/Y ratio). A decreased X/Y ratio reflects more pronounced trabeculations, with lower values indicating greater severity of non-compaction [9].

While various methods exist to assess myocardial appearance, the Jenni criteria are the most widely used in contemporary clinical practice (Table 1) [10]. These criteria rely on four specific echocardiographic observations, typically measured in the parasternal short-axis and apical views:

**Table 1.** **Jenni criteria**.

(i)	Bilayered myocardium with multiple, prominent trabeculations in end-systole
(ii)	Noncompacted-to-compacted (NC:C) ratio of >2:1
(iii)	Communication with the intertrabecular space was demonstrated with colour Doppler
(iv)	Absence of coexisting cardiac abnormalities

#### 1.2.2 Cardiac Magnetic Resonance (CMR) Imaging Criteria

In patients where the apex is difficult to visualize with TTE, CMR provides greater spatial resolution by imaging in all planes. To diagnose LVNC using CMR, clinicians typically utilize the Petersen criteria, which are applied across different imaging planes to evaluate myocardial structure. While echocardiography assesses the heart during systole, CMR measurements are obtained at the end of diastole, where a non-compacted-to-compact (NC:C) ratio >2.3 is required for a positive diagnosis [11]. To ensure a comprehensive assessment, this ratio is evaluated using two primary types of imaging orientations: Long-Axis (LAX) and Short-Axis (SAX) views (Fig. 2) [12].

**Fig. 2.**
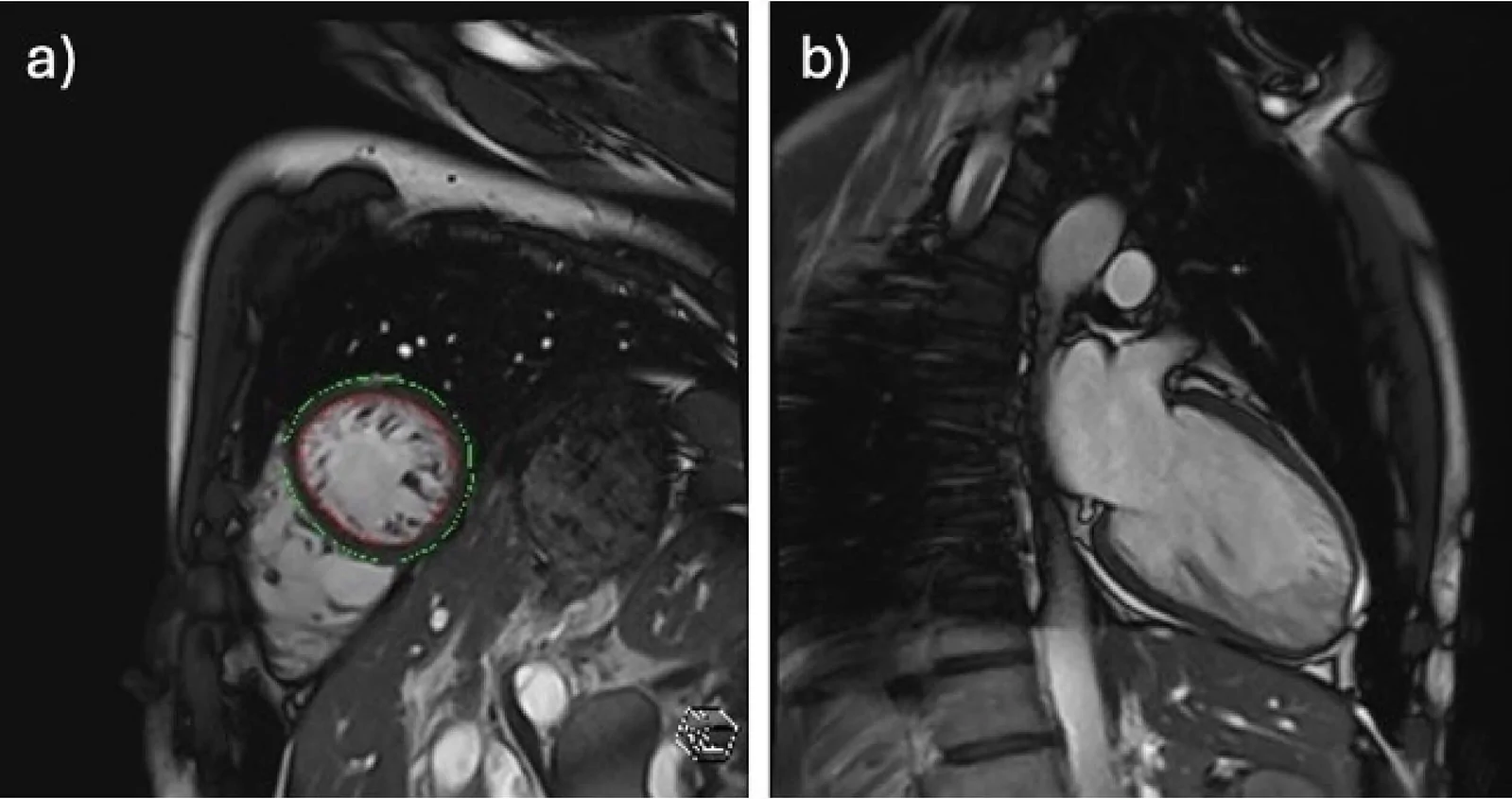
**CMR LVNC**. (a) Short-axis view with endocardial (red) and epicardial (green) contour tracings delineating the prominent trabeculations and deep intertrabecular recesses characteristic of LVNC. (b) Long-axis view further demonstrating the extent of myocardial non-compaction, with a total trabecular measurement of 22.8 mm. Together, these views yielded a NC:C ratio of 4.2:1, exceeding the diagnostic threshold of 2.3:1 established by Petersen’s criteria for LVNC. CMR, cardiac magnetic resonance imaging; LVNC, left ventricular noncompaction.

CMR provides exceptional diagnostic accuracy, boasting a sensitivity of 86% [11]. Similar to the use of colour Doppler in echocardiography, CMR black-blood imaging can assist in the diagnosis by identifying areas of high signal intensity within the trabeculae, which suggests stagnant blood flow [1].

## 2. Methods

This study used a single-center, combined retrospective and prospective cohort design to characterize the baseline clinical, imaging, electrophysiologic, and genetic features of adult patients with LVNC (Table 2). Data were managed and stored within a clinical registry maintained by the ICC clinic, University Hospital, London, Ontario, Canada. This report presents the baseline (cross-sectional) analysis of the registry; formal time-to-event analysis of clinical outcomes was not performed and is planned once sufficient prospective follow-up has accrued.

**Table 2.** **Data acquisition was tailored to the patient’s current clinical status**.

Prospective Cohort	For patients currently under the active care of physicians at the Inherited Arrhythmia Clinic, data will be collected prospectively during their regular clinical evaluations.
Retrospective Cohort	For patients not currently under active follow-up, a retrospective review of medical records will be conducted to capture historical clinical data and outcomes.

### 2.1 Data Collection and Longitudinal Follow-up

Patients were enrolled in the registry with baseline data collected at the time of LVNC diagnosis, including demographic characteristics, clinical presentation, and diagnostic imaging findings. Follow-up assessments were organized chronologically by year following enrollment, with follow-up 1 representing data collected approximately one year post-enrollment and follow-up 2 representing data collected approximately two years post-enrollment. Because attendance was variable, the interval between assessments varied among patients (see Limitations). Extracted data, including imaging findings, electrophysiologic reports, and major adverse cardiac events (MACE, defined for this registry as a composite of cardiovascular death, heart transplantation, appropriate ICD therapy, sustained ventricular arrhythmia, hospitalization for heart failure, and thromboembolic event), were integrated into the Research Electronic Data Capture (REDCap) database. The registry is designed to accrue data over a 10-year period; the present analysis reports only the baseline cross-section, and MACE incidence and time-to-event outcomes will be reported once adequate prospective follow-up has been collected.

Prospective patients are being monitored over a planned ten-year study period to assess disease evolution and clinical outcomes, while retrospective patients had their historical records reviewed for up to 15 years prior to enrollment; new patients diagnosed with LVNC are followed prospectively going forward.

### 2.2 Inclusion and Exclusion Criteria

Participants for this study are selected via a non-probability sampling method, targeting a specific population of adults with a confirmed diagnosis of LVNC.

To be eligible for inclusion, patients must be 18 years of age or older at the time of enrollment. For those assigned to the retrospective cohort, the confirmed diagnosis of LVNC must have been established between 2010 and 2020. The study population includes all sexes and comprises both a prospective cohort of patients currently under active follow-up at the ICC clinic and a retrospective cohort of patients no longer receiving active care at the facility.

Of 52 patients with an LVNC diagnosis followed at the ICC clinic during the study period who were assessed for eligibility, 50 met the inclusion criteria and were enrolled, comprising 38 prospective and 12 retrospective patients; the 2 remaining patients did not meet the inclusion criteria described below.

Patients were excluded if they did not meet the diagnostic criteria for LVNC or if they had a primary diagnosis of another cardiomyopathy (e.g., hypertrophic cardiomyopathy, dilated cardiomyopathy, arrhythmogenic right ventricular cardiomyopathy) without features that met the LVNC criteria. The exclusion is necessary to ensure that the longitudinal data collected specifically reflects the progression and clinical outcomes of LVNC rather than the confounding effects of secondary genetic cardiac pathologies. Patients with genetic mutations associated with cardiomyopathy were only included if they met imaging criteria for LVNC. Data collection included a baseline assessment including age at diagnosis; sex and gender; NYHA symptoms; history of syncope, palpitations and exertion symptoms; family history of SCD, LVNC, or genetic mutations linked to cardiomyopathy; or if the patients had a primary or secondary prevention ICD. The diagnostic imaging modality initially used was an echocardiogram, followed by CMR to evaluate for late gadolinium enhancement (LGE). Electrocardiograms were used to evaluate for T-wave abnormalities in multiple precordial leads and to correlate with the area of noncompaction. An exercise stress test (EST) to evaluate for arrhythmia provocation and genetic testing for inherited cardiomyopathy risk.

### 2.3 Genetic Testing

Genetic testing was obtained through two mechanisms, depending on cohort assignment. For patients in the prospective cohort, genetic testing was performed using the Adult Cardiomyopathy Panel (next-generation sequencing) at the CHEO Genetics Diagnostic Laboratory (Children’s Hospital of Eastern Ontario, Ottawa, Ontario), following referral through the LHSC Medical Genetics Program. Sequence variants were classified according to the American College of Medical Genetics and Genomics (ACMG)/Association for Molecular Pathology (AMP) criteria, ClinGen Sequence Variant Interpretation Working Group recommendations, and CHEO-specific internal guidelines. For patients in the retrospective cohort, genetic testing had been performed previously as part of prior clinical care at the discretion of the treating physician; as such, the specific panel and testing laboratory varied by patient and reflect standard clinical practice at the time of testing. Variants of uncertain significance (VUS) were analyzed and reported separately from pathogenic and likely pathogenic variants throughout this manuscript.

## 3. Results

### 3.1 Baseline Demographics

The study population consisted of 50 patients (females = 34), with a mean age of 48.9 ± 13.4 years, who had a confirmed diagnosis of LVNC and were followed at the ICC clinic. The patients were enrolled prospectively (n = 38) and retrospectively (n = 12) (Table 3). The diagnosis was established using echocardiography in 9 patients (18%), computed tomography (CT) in 1 patient (2%), and CMR in 40 patients (80%).

**Table 3.** **Baseline characteristics of the LVNC cohort (N = 50)**.

Characteristic	Value
Age at diagnosis, years (mean ± SD)	48.9 ± 13.4
Female sex, n (%)	34 (68%)
Male sex, n (%)	16 (32%)
Enrollment: prospective / retrospective, n (%)	38 (76%) / 12 (24%)
Diagnostic modality: CMR / TTE / CT, n (%)	40 (80%) / 9 (18%) / 1 (2%)
Prior cardiac arrest, n (%)	3 (6%)
Syncope, n (%)	10 (20%)
Palpitations, n (%)	16 (32%)
Family history of sudden cardiac death, n (%)	9 (18%)
NYHA class I / II / III / no heart failure, n (%)	4 (8%) / 2 (4%) / 2 (4%) / 42 (84%)
Baseline TTE available, n (%); mean LVEF	38 (76%); 51.8 ± 11.8%
Baseline CMR available, n (%); mean LVEF; median NC:C ratio (range)	44 (88%); 55.0 ± 9.7%; 4.0:1 (2.8–6.3:1)
12-lead ECG available, n (%)	48 (96%)
Genetic testing performed (of 38 prospective patients), n (%)	32 (84%)
Pathogenic/likely pathogenic variant identified (of 32 tested), n (%)	17 (53%)

NYHA, New York Heart Association; LVEF, left ventricular ejection fraction; ECG, electrocardiogram.

Within this cohort, 3 patients had experienced a prior cardiac arrest, 10 had a history of syncope, 16 reported palpitations, and 9 patients had a family history of sudden cardiac death. Based on the New York Heart Association (NYHA) functional classification, 4 patients were class I, 2 class II, and 2 class III. 42 patients did not have heart failure.

### 3.2 Echocardiography

Baseline TTE was available for 38. The mean LVEF was 51.8 ± 11.8%. The mean left ventricular mass indexed (LV_MI_) was 76.9 g/m^2^. The mean LV end-systolic volume index (LVESVi) was 37.7 ± 25.2 mL/m^2^, and the mean LV end-diastolic volume index (LVEDVi) was 69.97 ± 25.51 mL/m^2^.

Valvular findings in the cohort were predominantly mild or clinically insignificant. Several patients demonstrated no significant valvular abnormalities. Among those with abnormalities, the most common finding was mild mitral regurgitation, followed by mild tricuspid regurgitation. Trace regurgitation of the mitral, tricuspid, aortic, and pulmonic valves was also observed in a subset of patients. Where moderate hypertrabeculation was among the most common. 5 patients had regional wall motion abnormalities, manifesting as hypokinesis or akinesis of the apical and lateral walls corresponding to areas of maximal trabeculation, and 9 patients had global hypokinesis of the LV. Strain analysis was performed in 24 patients (63%), yielding a mean global longitudinal strain (GLS) of –16.18%. Of the remaining patients, 5 were unable to complete or provide adequate strain measurements, and 9 were excluded due to the unavailability of transthoracic echocardiography (TTE) images for analysis.

### 3.3 Cardiac Magnetic Resonance

Baseline CMR imaging was available for 44 patients, representing 88% of the cohort (44/50). All patients met CMR diagnostic criteria for LVNC based on the Petersen criteria (end-diastolic non-compacted to compacted myocardial ratio >2.3:1). The reported NC:C myocardial ratios ranged from 2.8:1 to 6.3:1 (median 4.0:1), substantially exceeding the Petersen diagnostic threshold in all cases.

The distribution of hypertrabeculation demonstrated a characteristic pattern (percentages calculated as a proportion of the 44 CMR-imaged patients): apical segments were involved in all cases (44/44, 100%), and lateral segments were affected in 32 patients (72.7%). Hypertrabeculation extended to the mid-ventricular segments in 19 patients (43.2%), with involvement of the anterior, anterolateral, inferolateral, and inferior mid-ventricular walls. Basal segment involvement was less common, observed in 3 patients (6.8%), primarily affecting the lateral and inferolateral walls.

Marked thinning of the compacted myocardial layer was frequently observed, with a maximal thickness of 3–7 mm in affected segments. The lateral and inferolateral walls were most involved, with compacted myocardial thickness ranging from 0.4 cm to 0.7 cm in these regions.

LV volumes and systolic function showed the following parameters: LV ejection fraction (LVEF) was 55.0 ± 9.7%, LVEDVi was 91.7 ± 22.6 mL/m^2^, and LVESVi was 44.3 ± 17.16 mL/m^2^.

Late gadolinium enhancement (LGE) was detected in 6 patients (13.6%), with patterns consistent with non-ischemic myocardial fibrosis. Enhancement distributions included mesocardial to subepicardial involvement of the mid anterolateral and inferolateral walls, patchy enhancement in hypertrophied basal segments, and extensive apical and mid-cavity free wall involvement. One patient demonstrated enhancement in a pattern consistent with prior ventricular septal defect repair. No left ventricular thrombi were identified.

### 3.4 Electrocardiographic

12-lead electrocardiographic assessment was performed in 48 patients (96% of the cohort).

Basic electrocardiographic parameters demonstrated the following: median heart rate 67 beats per minute (bpm) (IQR 58.5–76 bpm), median QRS duration 94 ms (IQR 88–113 ms), and median corrected QT interval (QTc) 423.5 ms (IQR 403.5–445 ms).

T-wave abnormalities constituted the most frequent finding, observed in 10 of the 48 patients with ECG data (20.8%). Premature ventricular complexes (PVCs) were documented in 4 patients (8.3%) during routine ECG acquisition. Additional electrocardiographic findings, expressed as a proportion of the 48 patients with ECG data, included septal infarction pattern in 6 patients (12.5%), low-voltage patterns in 2 patients (4.2%), left bundle branch block in 7 patients (14.6%), and right bundle branch block in 3 patients (6.3%). Anterior infarction pattern was identified in 1 patient (2.1%), and first-degree atrioventricular block was also noted in 1 patient (2.1%). Because CMR with late gadolinium enhancement was not available for all patients, ECG-based infarction patterns (Q waves) serve as an indirect, non-confirmatory marker of prior myocardial injury rather than a CMR-verified diagnosis.

### 3.5 Exercise Stress Tests

Exercise stress testing (EST) was performed in 14 patients (28%) using the Bruce Protocol. The median exercise duration was 9.0 minutes (IQR 6.46–10.0 minutes), with a mean maximum heart rate of 143.7 bpm. Arrhythmias were common during testing: PVCs were observed in 10 patients (71%), including 1 patient with trigeminy and 1 with non-sustained ventricular tachycardia (NSVT). Additionally, new-onset intraventricular conduction delay (NIVCD) was observed in 3 patients (21%).

### 3.6 Holter Monitoring

Holter monitoring was performed in 13 patients, all of whom showed sinus rhythm. Ectopic burden was low in 12 of 13 patients, with rare isolated premature atrial complexes (PACs) and PVCs accounting for <1% of total beats. One patient exhibited symptomatic frequent PVCs (22% burden) of a single morphology. No sustained ventricular tachycardia, atrial fibrillation, or significant pauses were observed. No clinically significant arrhythmias were identified in the remaining patients.

### 3.7 Genetic Basis

Genetic testing identified multiple variants of varying clinical significance among patients with confirmed LVNC (Fig. 3). Of the prospective cohort (n = 38), genetic results were available for 32 patients (84%). A pathogenic or likely pathogenic (P/LP) variant, per ACMG/AMP classification (see Methods), was identified in 17 of 32 tested patients (53%). The remaining 15 patients (47%) had no P/LP variant identified (variants of uncertain significance, discussed separately below, are not counted as positive findings). Among patients with a P/LP variant, the most common gene involved was TTN (n = 11, 34% of the tested cohort), followed by Filamin-C (FLNC) (n = 2, 6%), Myosin-Binding Protein C (MYBPC3) (n = 2, 6%), Myosin Heavy Chain 7 (MYH7) (n = 2, 6%), and RNA Binding Motif Protein (RBM20) (n = 2, 6%).

**Fig. 3.**
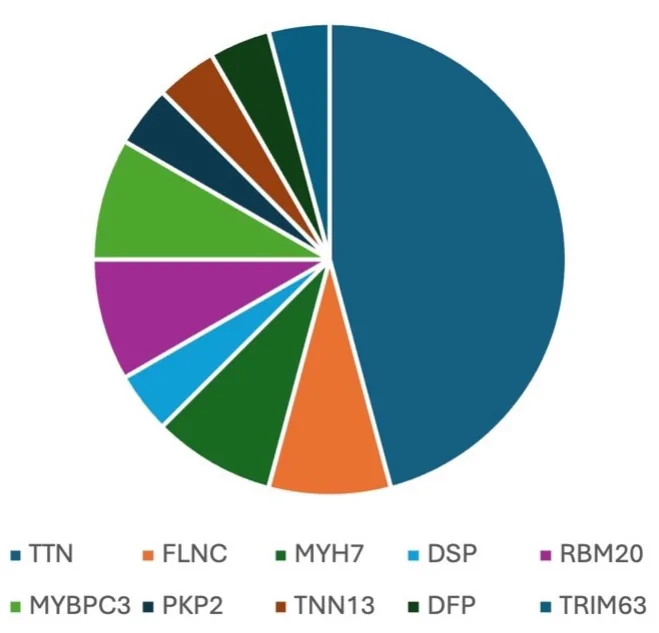
**Prevalence of pathogenic/likely pathogenic genetic variants among the 32 genetically tested patients: TTN 34% (n = 11); FLNC, MYBPC3, MYH7, and RBM20 each 6% (n = 2)**. Variants of uncertain significance (DSP, PKP2, TNNI3, and others) were each identified in 1 patient (3%) and are reported separately from pathogenic/likely pathogenic variants. FLNC, Filamin-C; MYBPC3, Myosin-Binding Protein C; TNNI3, Cardiac Troponin I; MYH7, Myosin Heavy Chain 7; DSP, Desmoplakin; RBM20, RNA Binding Motif Protein; PKP2, Plakophilin-2; TRIM63, Tripartite Motif Containing 63.

Several patients harboured more than one P/LP variant; therefore, gene-specific counts above are not mutually exclusive. Separately from the P/LP variants above, variants of uncertain significance (VUS) were identified in individual patients, including DSP, PKP2, TNNI3, and others; because VUS carry no established diagnostic or prognostic weight, they are reported descriptively only and are not included in the pathogenic/likely pathogenic counts or in the gene-specific risk discussion below.

### 3.8 Medical Therapy

Medical therapy was initiated and maintained based on guideline-directed medical therapy (Fig. 4).

**Fig. 4.**
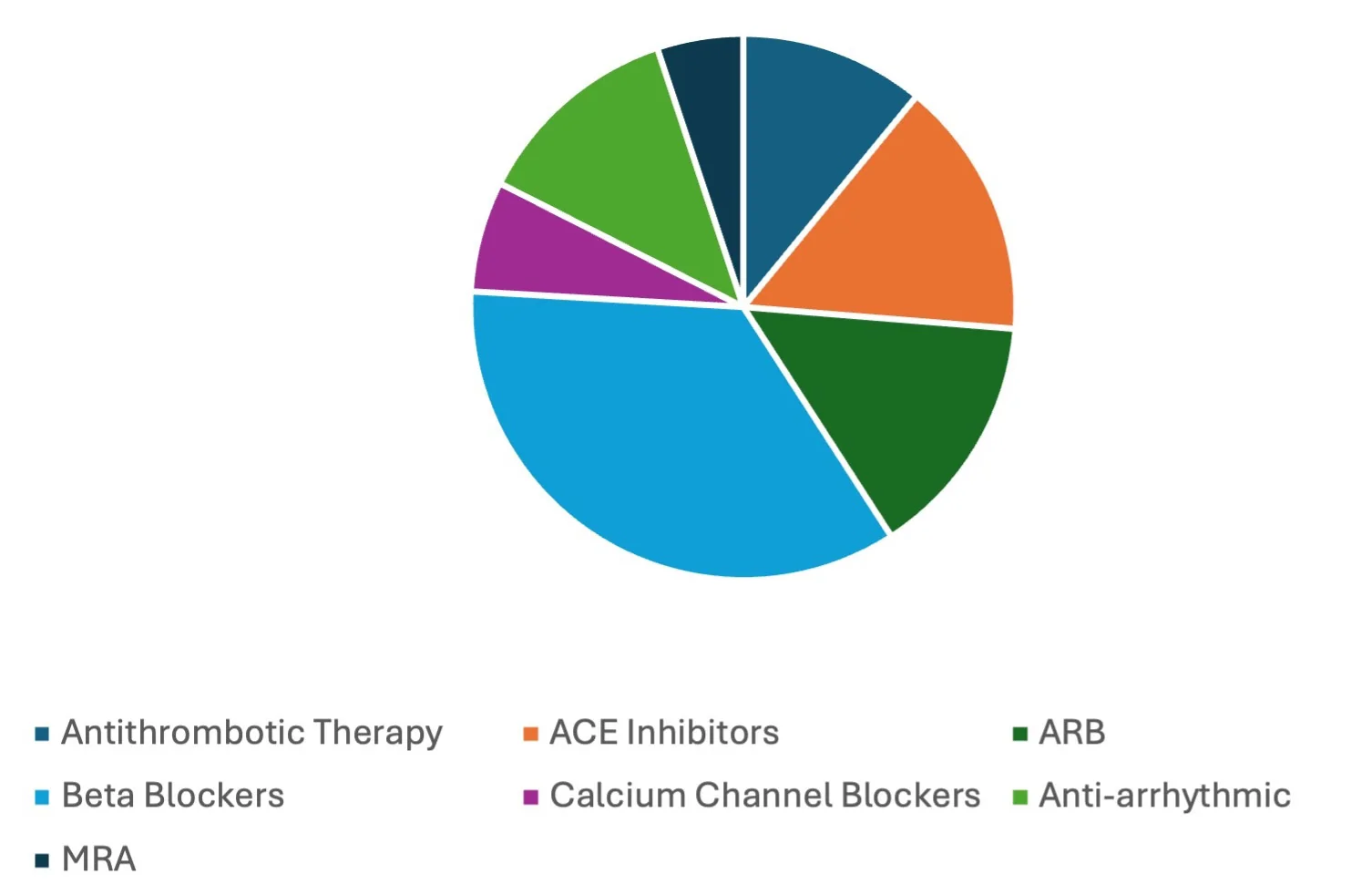
**Medical therapy from baseline assessment of a 50 patient cohort**. 15% of patients are on antithrombotic therapy/oral anticoagulation, with acetylsalicylic acid being the most common. 21% on ACE inhibitors. 20% on angiotensin receptor blockers (ARBs). 48% on beta blockers, with bisoprolol being the most common. 9% on calcium channel blockers. 17% on anti-arrhythmic. 7% on mineralocorticoid receptor antagonists (MRAs).

### 3.9 Follow-up-1 Results

Attendance for structured follow-up was variable and incomplete across modalities. Of the 50 patients (38 prospective, 12 retrospective), a follow-up-1 assessment was completed in 39 (78%); 4 patients were formally lost to follow-up, and 7 had not yet reached their follow-up window. Among the 29 prospective patients completing follow-up 1 within their protocol-defined window, the median time to assessment was 9.8 months (IQR 7.5–14.6); intervals for retrospective patients instead reflect the variable spacing of their existing clinical follow-up rather than a fixed protocol window. Follow-up-2 data were available for 29 of 50 patients (58%), representing 42% attrition by the second follow-up interval. Because attendance for each modality (TTE, ECG, CMR, EST, Holter) differed within each follow-up interval, the denominators below vary by modality and are stated explicitly; the following results should be interpreted as a description of the subset tested rather than the full cohort, and no paired statistical comparison between follow-up 1 and follow-up 2 was performed in this preliminary descriptive analysis.

Of the 50 patients eligible for follow-up 1, 16 patients (32%) underwent TTE with LVEF of 51.1 ± 10.9%. Strain analysis was completed in 10 patients (62%), with a mean GLS of –17.02%.

Electrocardiograms were available for 32 patients (64%). T-wave abnormalities were present in 6 patients (19% of those with ECGs available). The median QRS duration was 96 ms (IQR 87.5–112.5 ms), the median heart rate was 64 bpm (IQR 57.7–77 bpm), and the median QTc interval was 438 ms (IQR 399.5–466.5 ms). Notable outliers included one patient with markedly prolonged QRS duration (168 ms, suggestive of complete bundle branch block or ventricular conduction delay), one patient with severe bradycardia (41 bpm), one with tachycardia (134 bpm), and two patients with extreme QTc intervals (255 ms and 522 ms), the latter indicating significant risk for ventricular arrhythmias. PVCs were observed in 4 patients. Other findings included first-degree atrioventricular block, right bundle branch block, atrial fibrillation with rapid ventricular response, atrial-paced rhythm with prolonged atrioventricular conduction and left axis deviation with nonspecific intraventricular conduction delay, and low-voltage QRS complexes.

Seven patients (14%) completed an EST, with a mean exercise duration of 10.8 ± 1.7 minutes. On average, patients achieved 92.3% of their target heart rate. Of the 6 patients with a documented reason for test termination (one patient had two reasons), this included shortness of breath in 1 patient (16.7%), fatigue in 1 patient (16.7%), reaching target heart rate in 4 patients (66.7%), and other reasons in 1 patient (16.7%). Three patients (50%) demonstrated PVC.

Two patients (4%) underwent CMR, where LVEF were 57% and 37%, respectively. Both patients had increased hypertrabeculations involving mid to apical segments, with one having significant arrhythmia, which degrades the assessment.

Six patients had a Holter monitor assessment, with the predominant rhythm sinus and rare PVCs and PACs; the longest was 6 beats of atrial ectopy at 115 BPM.

### 3.10 Follow-up-2 Results

Of the 29 patients with available follow-up 2 data, echocardiography was performed in 17 patients, representing 58% of the cohort. The mean LVEF was 56 ± 6%. Strain analysis was completed in 10 patients, with a mean GLS of –15.07%.

Electrocardiograms were available for 24 patients (82%). Three patients demonstrated T-wave abnormalities in multiple precordial leads. The median QRS duration was 98 ms (IQR 83–110 ms), the median heart rate was 62 bpm (IQR 54.5–75.5 bpm), and the median QTc interval was 413.5 ms (IQR 383–437 ms).

Two patients underwent CMR, with a mean LVEF of 59 ± 2%. Hypertrabeculation was noted, with an NC:C ratio estimated of 3.7:1.

Two patients completed EST, with an average exercise duration was 10.1 minutes. Both patients achieved their target heart rate. One patient exhibited PVC during testing.

Seven patients underwent Holter monitoring; sinus rhythm was present in all, with one demonstrating a bundle branch block pattern. Atrial and ventricular ectopy burden was low in 5 of the remaining 6 patients (excluding the one with a bundle branch block pattern), with rare, isolated PACs and PVCs accounting for less than 1 percent of total beats. One patient demonstrated a higher PVC burden (14%).

## 4. Discussion

In this single-center cohort of 50 patients with LVNC, we describe a broad range of clinical, imaging, electrophysiologic, and genetic characteristics. The mean age at diagnosis in the fifth decade of life aligns with published data showing median ages at diagnosis ranging from 41 to 52 years in adult cohorts [13].

Additionally, the cohort demonstrated a 68% female predominance, which diverges from most published LVNC registries [4,13]. Casas et al. [13] (n = 437) reported 44% female, van Waning et al. [14] (n = 327) reported 53% female, and Andreini et al. [15] (n = 113) reported 38% female. Several factors may explain this discrepancy, such as referral bias at a tertiary ICC clinic may select for symptomatic patients with palpitations, a symptom more frequently reported by women. Additionally, the relatively preserved mean LVEF (51.8% by TTE, 55% by CMR) may reflect known sex-based differences in LVNC phenotype: women with LVNC tend to present with higher LVEF and less LV dilation than men, potentially representing a milder phenotypic spectrum increasingly detected with wider CMR utilization [14]. The high proportion of CMR-based diagnoses (80%) may also contribute, as CMR detects hypertrabeculation in individuals who might not meet echocardiographic criteria, and population-based CMR studies have shown that up to 14.8% of healthy individuals meet at least one LVNC criterion [16]. Whether the female predominance reflects true epidemiologic variation, referral patterns, or diagnostic modality sensitivity warrants investigation in larger multicenter registries.

### 4.1 Genetic Findings

In this prospective cohort of 38 patients with confirmed LVNC, genetic testing identified a pathogenic or likely pathogenic (P/LP) variant in 53% (17/32) of tested individuals, which aligns with the upper range of the 17–41% typically reported in symptomatic LVNC cohorts [2,17,18,19]. The most striking finding was the high prevalence of TTN variants (n = 11, 34% of tested patients), substantially exceeding the 13% frequency reported in most LVNC cohorts and consistent with the strong association between TTN truncating variants and LVNC (odds ratio 40.7) [18,20]. TTN variants were associated with reduced systolic function (mean EF = 44%), consistent with published data showing that TTN truncating variant carriers experience lower LVEF and increased heart failure events compared to non-carriers. These findings underscore the critical role of titin-mediated pathomechanisms in LVNC pathogenesis and support the prioritization of TTN in genetic counseling for affected families.

Beyond TTN, the cohort also included small numbers of patients with FLNC (n = 2) and RBM20 (n = 2) variants. In larger published series, these genes are associated with disproportionate arrhythmic and heart failure risk independent of LV dysfunction severity, and would generally warrant closer surveillance. However, with only two patients per gene in this cohort, we cannot independently confirm a gene-specific risk signal here, and we do not intend these observations to imply a specific management recommendation for this registry’s patients beyond standard genotype-informed counseling; the published associations [21,22,23] for RBM20 are summarized below for context [14,18,24]. MYBPC3 and MYH7 variants were each identified in two patients, consistent with sarcomeric genes accounting for 20–32% of genetically explained LVNC cases [2,13,17].

Several patients harbored variants in multiple genes, reflecting the 21% prevalence of complex genotypes associated with lower ejection fractions and increased heart failure risk [25]. The substantial proportion of patients without an identified pathogenic or likely pathogenic variant (47%) suggests that additional genetic mechanisms, including novel genes or non-coding variants, may contribute to LVNC pathogenesis in this population, though a proportion may also reflect a morphologic trait without an underlying monogenic cause [26]. The genetic heterogeneity observed in this cohort underscores the importance of comprehensive gene panel testing in LVNC patients. Given the small number of patients per gene in our cohort, we cannot independently validate genotype-specific risk stratification from these data; rather, we note that published literature on FLNC and RBM20 supports closer surveillance where these variants are found, and this warrants confirmation in larger, adequately powered cohorts before informing management decisions here.

### 4.2 Diagnostic Imaging

Echocardiography remains the most common initial diagnostic strategy, due to its low cost, widespread availability, and accessibility [27]. However, within our subset of patients, 40 patients (80%) had their diagnoses established using CMR, reflecting the increasing reliance on advanced imaging modalities for the evaluation of LVNC. The diagnostic criteria for LVNC include the most widely used criterion, an NC:C ratio >2:1 measured at end-diastole. However, there are currently no universally accepted diagnostic criteria or guidelines for LVNC [1]. CMR is increasingly recognized as the diagnostic gold standard, offering superior spatial resolution and comprehensive tissue characterization of noncompacted and compacted myocardium [28]. Additionally, the ability of CMR to identify the presence and extent of LGE as a surrogate marker of myocardial fibrosis is also employed to determine the extent of LV scarring.

### 4.3 Echocardiogram

In this cohort, baseline TTE showed a mean LVEF of 51.8%, within the low-normal range, suggesting that many patients had preserved or only mildly reduced systolic function at presentation. This aligns with the known heterogeneity of LVNC, where patients can range from asymptomatic with normal function to those with advanced systolic dysfunction (Fig. 5) [1]. The relatively wide standard deviation highlights this variability and supports that LVNC does not necessarily result in impaired contractility. The observed LV volumes, including a mean LVEDVi of 69.9 mL/m^2^ and LVESVi of 37.7 mL/m^2^, suggest mild chamber remodelling in some patients, which may reflect early or subclinical disease progression rather than overt dilated cardiomyopathy. Valvular abnormalities were largely mild or clinically insignificant, consistent with LVNC being primarily a myocardial disorder rather than presenting with valvular pathology.

**Fig. 5.**
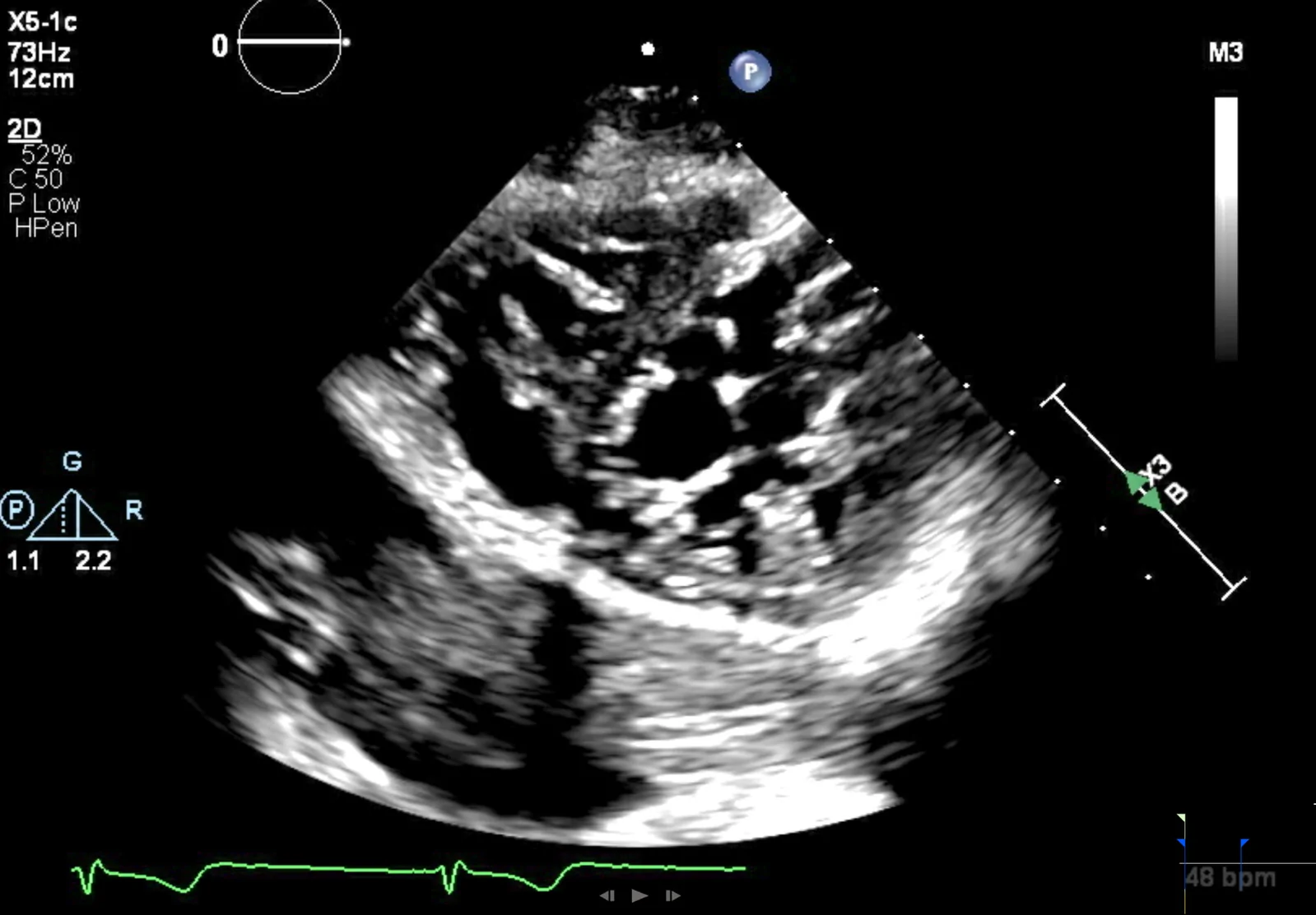
**TTE examination of a patient with LVNC**. Parasternal short axis, 2D, diastole. Apical-lateral hypertrabeculation of the left ventricle. Ejection Fraction 55–60%.

Strain analysis was performed in 24 patients, of whom 4 were unable to obtain adequate measurements. Among those assessed, the mean GLS was −16.18%, falling within the borderline range (−16% to −18%), consistent with subclinical myocardial dysfunction [25]. This finding aligns with prior studies demonstrating impaired GLS in LVNC patients even in the setting of preserved LVEF, reflecting the superior sensitivity of strain imaging in detecting early LV dysfunction antecedent to overt systolic impairment [29]. However, strain analysis in LVNC patients can be technically challenging due to poor acoustic windows and suboptimal image quality resulting from the extensive trabeculations and deep intertrabecular recesses that characterize the condition, which may limit endocardial border definition and tracking accuracy. The degree of GLS impairment has been shown to correlate with reduced LVEF and carries prognostic implications, potentially identifying patients at risk for progression to overt LV dysfunction who may warrant closer surveillance [29]. Additionally, impaired strain parameters, particularly apical circumferential strain and twist, have been shown to associate with cardiovascular events independent of LVEF, suggesting that strain analysis may complement risk stratification beyond traditional functional parameters [26].

Overall, these findings indicate that most patients with LVNC in this cohort had preserved systolic function and minimal secondary valvular involvement at baseline, underscoring the importance of longitudinal follow-up to assess for disease progression and functional decline.

### 4.4 Electrocardiogram

Arrhythmic manifestations in this cohort included syncope and palpitations, with a subset of patients having a family history of cardiac arrest and other cardiac-related events. Several individuals were classified as higher risk for SCD, necessitating careful rhythm monitoring and individualized risk stratification.

ECG abnormalities are present in 80–90% of LVNC patients, with entirely normal ECGs found in only 10–13% of cases [1]. In the comprehensive analysis by Steffel et al. [30] of 78 LVNC patients at initial diagnosis, only 10 subjects (13%) had entirely normal ECGs, with the majority demonstrating intraventricular conduction delay, LV hypertrophy by voltage criteria, and repolarization abnormalities. However, ECG findings dramatically vary depending on cohort, and there are thus no specific ECG findings correlated with LVNC [31].

In the present cohort, T-wave abnormalities were the most frequent finding, 20.8%, though this is lower than the up to 87% prevalence reported in some series. Repolarization abnormalities, including T-wave inversion and ST-segment changes, are among the most common ECG findings in LVNC and may reflect underlying myocardial dysfunction. Intraventricular conduction abnormalities were substantial, with left bundle branch block (LBBB) in 14.6% and right bundle branch block (RBBB) in 6.3% within the ECG cohort. LBBB is particularly significant as it demonstrated a striking overlap with reduced systolic LV function and LV/left atrial dilation and has been identified as a predictor of fatal arrhythmic events in systematic reviews of LVNC patients.

Evidence of prior myocardial injury was documented in 14.6% of patients (septal pattern 12.5%, anterior pattern 2.1%), likely reflecting the thromboembolism risk from stagnant blood flow within deep intertrabecular recesses. These infarction patterns may also indicate areas of myocardial fibrosis or scar, which serve as arrhythmogenic substrates. PVCs were documented in 8.3% during routine ECG, though this represents only a snapshot of ventricular ectopy burden, and extended rhythm monitoring would likely reveal a higher prevalence of ventricular arrhythmias in this population.

The electrocardiographic profile demonstrates the nonspecific yet clinically significant nature of ECG abnormalities in LVNC. While no pathognomonic pattern exists, the range of findings reflects the underlying substrate predisposing to heart failure, arrhythmias, and sudden cardiac death.

### 4.5 Cardiac Magnetic Resonance Imaging

These patients met established Petersen diagnostic criteria, with NC:C ratios substantially exceeding the diagnostic threshold of 2.3:1 (Fig. 6). The distribution of hypertrabeculation, severity of morphological changes, and associated functional abnormalities observed in this cohort align with previously reported patterns in LVNC, while also highlighting important considerations for clinical management and risk stratification.

**Fig. 6.**
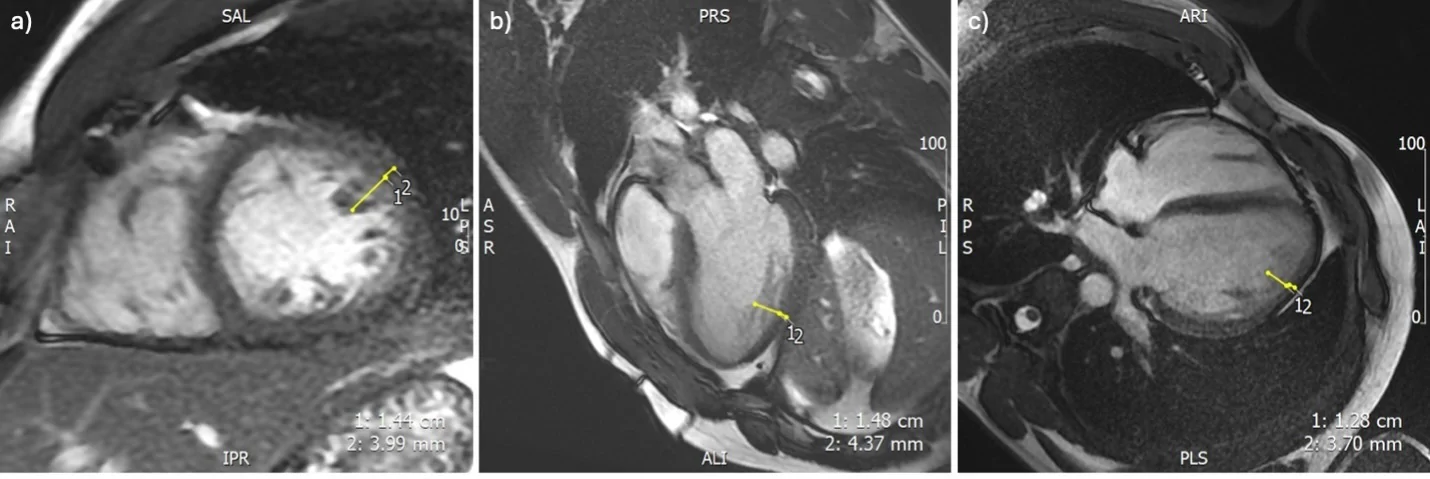
**CMR across three orthogonal planes. Measurements denote the non-compacted trabecular (label 1) and compacted (label 2) myocardial layers, with the yellow calipers placed at end-diastole per standard LVNC quantification**. (a) Short-axis view (SAL–IPR): Cross-sectional appearance of the left ventricle with prominent hypertrabeculation along the lateral wall. Non-compacted layer: 1.44 cm (14.4 mm); compacted layer: 3.99 mm. NC/C ratio: 3.61. (b) Vertical long-axis (two-chamber) view (PRS–ALI): Longitudinal extent of trabeculations is best appreciated here, most pronounced at the apical and inferolateral segments. Non-compacted layer: 1.48 cm (14.8 mm); compacted layer: 4.37 mm. NC/C ratio: 3.39. (c) Horizontal long-axis (four-chamber) view (ARI–PLS): A third imaging plane confirming trabecular prominence along the lateral free wall. Non-compacted layer: 1.28 cm (12.8 mm); compacted layer: 3.70 mm. NC/C ratio: 3.46.

The anatomical distribution of hypertrabeculation in our cohort demonstrates the characteristic predilection for the apical and lateral segments, as consistently reported in the LVNC literature [1]. Apical involvement was universal in our series, with lateral segment involvement observed in 72.7% (32/44) of CMR-imaged patients. This distribution pattern reflects the embryological development of the left ventricle.

The high frequency of lateral involvement (72.7%) in our cohort is noteworthy and suggests more extensive disease than might be appreciated by echocardiography alone, which may have limited visualization of these segments. The relatively infrequent basal involvement (6.8%) is consistent with the compaction gradient, as these segments typically complete compaction earliest in development. The predominance of lateral and inferolateral wall involvement across all ventricular levels underscores the importance of a comprehensive assessment of these regions during CMR (Fig. 7).

**Fig. 7.**
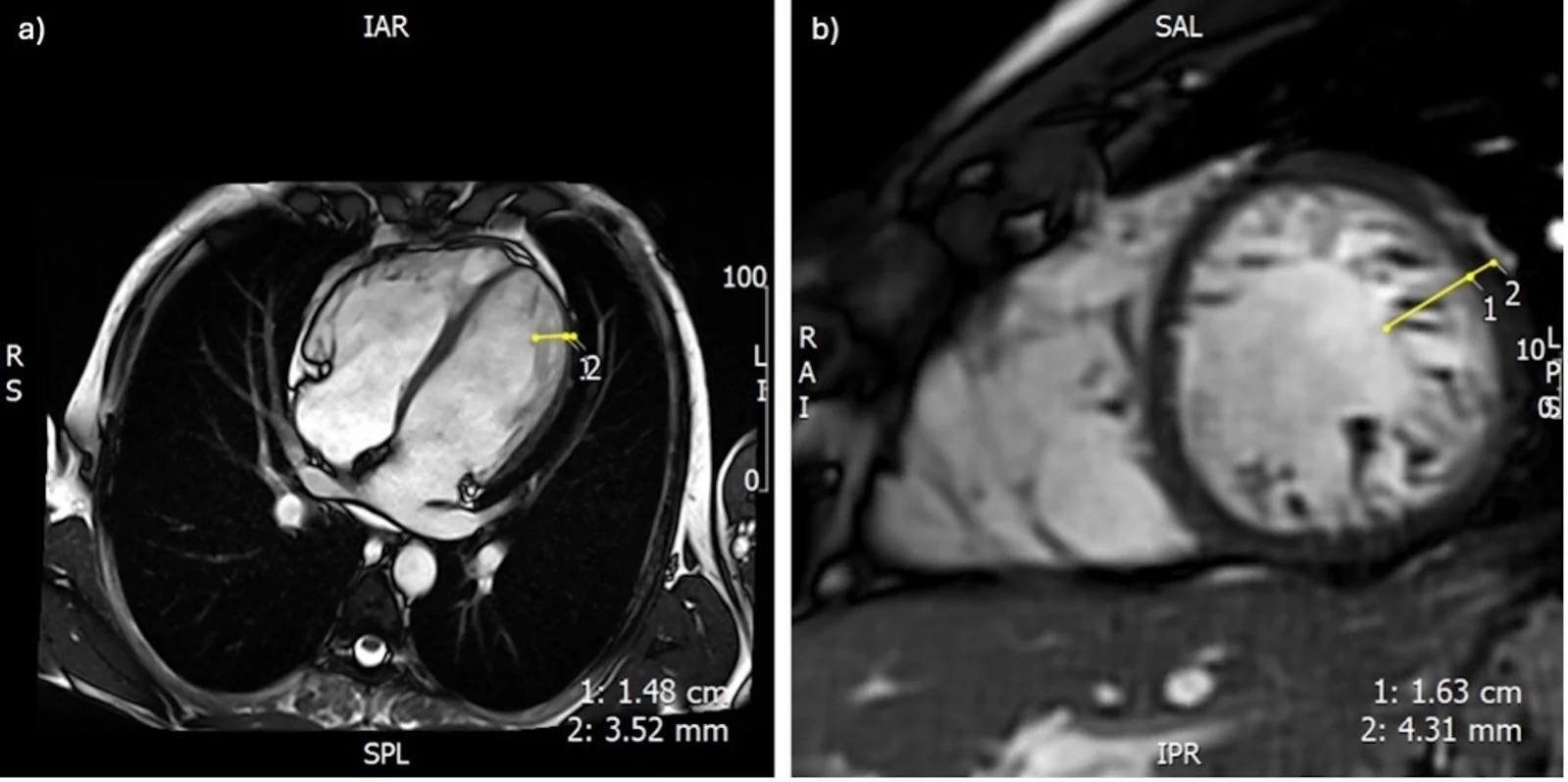
**CMR characterizing the extent and distribution of left ventricular trabeculation. Measurements denote the non-compacted trabecular (label 1) and compacted (label 2) myocardial layers**. (a) Axial/transverse (four-chamber equivalent) view (IAR–SPL): Demonstrates the global extent of non-compaction from a transverse perspective, with trabeculations most conspicuous along the lateral wall near the mid-ventricular level. Non-compacted layer: 1.48 cm (14.8 mm); compacted layer: 3.52 mm. NC/C ratio: 4.20. (b) Short-axis view (SAL–IPR): Mid-ventricular level showing the most pronounced trabecular thickness across all measured planes, concentrated at the anterolateral and lateral segments. Non-compacted layer: 1.63 cm (16.3 mm); compacted layer: 4.31 mm. NC/C ratio: 3.78.

### 4.6 Morphological Severity and Myocardial Thinning

The wide range of non-compacted-to-compact ratios observed (2.8:1 to 6.3:1) reflects the morphological heterogeneity of LVNC. The median ratio of 4.0:1 suggests that most patients in our cohort exhibited moderate-to-severe morphological changes.

The marked thinning of the compacted myocardial layer (measuring as little as 3–4 mm in some patients) raises important clinical concerns. This degree of thinning may compromise myocardial structural integrity and contribute to systolic dysfunction, ventricular arrhythmias, and adverse remodelling. Notably, the lateral and inferolateral walls demonstrated both the most severe thinning and the highest trabeculation burden, suggesting regional variability in morphological severity.

### 4.7 Clinical Course and Management

The recommended management of LVNC requires a comprehensive, individualized approach incorporating multimodal diagnostic imaging, systematic risk stratification based on ventricular function and arrhythmic substrate, and phenotype-directed treatment strategies. LVEF is the primary prognostic determinant. Patients with normal LV size and function undergo clinical monitoring, whereas symptomatic patients with LV dilation and dysfunction or hypertrophy are managed according to phenotype [13]. Arrhythmic risk assessment should incorporate clinical history (syncope, family history of sudden death), ambulatory monitoring findings (nonsustained VT), and imaging markers, including LGE [1,3]. Holter monitoring is recommended as part of clinical monitoring in LVNC patients, though the specific frequency is not firmly established in formal guidelines. Expert reviews suggest yearly Holter monitoring “appears to be essential” to screen for asymptomatic arrhythmias, given the high arrhythmia burden (ventricular tachyarrhythmias in >20% of patients) [3,4].

The current recommendations for treatment follow the international guidelines of heart failure management [32]. Treatment usually requires a combination of beta-blockers, ACE inhibitors/ARBs, diuretics, and aldosterone antagonists, as observed in our cohort. ICDs are recommended in those with EF<35% or those with previously life-threatening arrhythmias. One of the patients in our cohort population received an ICD implant due to LVEF 30–35%, narrow QRS. Another patient receives an upgrade from an ICD to a cardiac resynchronization therapy defibrillator (CRT-D) due to heart failure with a mid-range ejection fraction of 40–45% and polymorphic ventricular tachycardia (PMVT). Additionally, 12% of patients with LVNC progress to end-stage heart failure requiring transplantation, though this varies significantly by phenotype [31]. Baseline LV dilation (LVEDD z-score >4) and moderate to severe LV dysfunction were independently associated with progression to death or transplantation**, **with transplantation-free survival of only 38% in this high-risk group [33].

## 5. Clinical Implications

As a cross-sectional baseline description, this registry cannot establish causal or prognostic relationships; the associations noted above (imaging distribution, functional status, arrhythmic burden, and genetic findings) are descriptive and hypothesis-generating. They nonetheless suggest that CMR adds diagnostic value where echocardiographic windows are limited, that systematic rhythm monitoring is warranted given the heterogeneity in ectopic burden observed, and that genetic testing has value for diagnostic confirmation and cascade family screening. Confirming whether these baseline features predict arrhythmic or heart failure events will require the planned prospective follow-up of this cohort.

## 6. Limitations

A significant methodological limitation of this study pertains to the follow-up assessment structure. Under the implemented protocol, if a patient failed to attend their scheduled visit during the first-year post-baseline, the Follow-up 1 assessment was coded as missing data, and any subsequent attendance in later years was designated as Follow-up 2. This sequential classification system introduces several analytical challenges. This method generates considerable heterogeneity in inter-assessment intervals across the patient cohort, as the temporal spacing between consecutive follow-ups varies substantially depending on individual attendance patterns. This variable timing may compromise the comparability of longitudinal trajectories between participants, particularly when examining time-dependent outcomes or disease progression rates.

Additionally, given the modest cohort size, this data attrition further diminishes statistical power and may introduce selection bias if attendance patterns correlate with clinical or demographic characteristics, ultimately limiting the study’s capacity to detect meaningful longitudinal effects.

## 7. Conclusion

This baseline analysis of a single-center adult LVNC registry demonstrates substantial phenotypic, morphologic, and genetic heterogeneity: most patients had preserved or mildly reduced LV systolic function at diagnosis, hypertrabeculation was consistently apical and often lateral on CMR, and a pathogenic or likely pathogenic variant—most often in TTN—was identified in roughly half of genetically tested patients. As a cross-sectional description without formal outcome or time-to-event analysis, these findings are hypothesis-generating rather than prognostic. The key message for readers is that this cohort is now well-characterized at baseline and positioned for the prospective follow-up needed to determine which of these clinical, imaging, and genetic features actually predict arrhythmic events, heart failure progression, and other adverse outcomes in LVNC.

## Data Availability

The datasets generated and analyzed during the current study are not publicly available because they contain identifiable and sensitive patient information collected as part of an ongoing institutional registry at London Health Sciences Centre. De-identified data may be made available from the corresponding author upon reasonable request and with approval from the appropriate institutional ethics board, in accordance with institutional and privacy regulations.

## Abbreviations

AHA, American Heart Association; ARB, Angiotensin Receptor Blocker; ACE, Angiotensin-Converting Enzyme; ACMG, American College of Medical Genetics and Genomics; AMP, Association for Molecular Pathology; BPM, Beats Per Minute; CMR, Cardiac Magnetic Resonance; CRT-D, Cardiac Resynchronization Therapy Defibrillator; CT, Computed Tomography; DCM, Dilated Cardiomyopathy; DSP, Desmoplakin; ECG, Electrocardiogram; ESC, European Society of Cardiology; EST, Exercise Stress Test; FLNC, Filamin-C; GLS, Global Longitudinal Strain; HCM, Hypertrophic Cardiomyopathy; ICC, Inherited Cardiac Conditions; ICD, Implantable Cardioverter-Defibrillator; IQR, Interquartile Range; LAX, Long-Axis; LGE, Late Gadolinium Enhancement; LV, Left Ventricular/Left Ventricle; LVEDD, Left Ventricular End-Diastolic Diameter; LVEDVi, Left Ventricular End-Diastolic Volume Index; LVEF, Left Ventricular Ejection Fraction; LVESVi, Left Ventricular End-Systolic Volume Index; LVMI, Left Ventricular Mass Index; LVNC, Left Ventricular Noncompaction; MACE, Major Adverse Cardiac Events; MYBPC3, Myosin-Binding Protein C; MYH7, Myosin Heavy Chain 7; NC:C, Non-Compacted to Compacted (ratio); NIVCD, New-onset Intraventricular Conduction Delay; NSVT, Non-Sustained Ventricular Tachycardia; NYHA, New York Heart Association; PAC, Premature Atrial Complex; PKP2, Plakophilin-2; PMVT, Polymorphic Ventricular Tachycardia; PSAX, Parasternal Short-Axis; PVC, Premature Ventricular Complex/Contraction; QTc, Corrected QT Interval; RBM20, RNA Binding Motif Protein; REDCap, Research Electronic Data Capture; RWT, Relative wall thickness; SAX, Short-Axis; SCD, Sudden Cardiac Death; TNNI3, Cardiac Troponin I; TTN, Titin; TTE, Transthoracic Echocardiography; VT, Ventricular Tachycardia.
